# Video-Assisted Debriefing for Non-Technical Skills Training in Emergency Medicine Clerkships: A Mixed-Methods Feasibility Study

**DOI:** 10.2147/AMEP.S628951

**Published:** 2026-08-27

**Authors:** Khalid Bashir, Amani A Al-Rajhi, Amr Elmoheen, Salman Riaz, Abdulla A Al-Yousuf

**Affiliations:** 1 Clinical Department, College of Medicine, QU Health, Qatar University, Doha, Qatar; 2 Emergency Department, Hamad General Hospital, Hamad Medical Corporation, Doha, Qatar; 3 ITQAN Clinical Simulation and Innovation Center, Hamad Medical Corporation, Doha, Qatar

**Keywords:** non-technical skills, simulation, video-assisted debriefing, situational awareness, emergency medicine, medical education, mixed methods, feasibility study

## Abstract

**Objective:**

Non-technical skills (NTS) are essential for safe emergency care but are inconsistently taught in undergraduate curricula. Video-assisted debriefing (VAD) may support reflection on team performance, but its feasibility within routine clerkships is underexplored. This study evaluated the feasibility of integrating VAD into a mandatory emergency medicine clerkship and descriptively explored learner perceptions and observed NTS performance.

**Methods:**

A convergent mixed-methods feasibility study was conducted within an 8-week final-year emergency medicine clerkship in Qatar (N=66). Weekly simulation sessions included structured NTS teaching and VAD. Feasibility was predefined as: participation ≥80%, survey completion ≥70%, and ≥50% of recordings technically suitable for faculty assessment using the Simulation Performance Assessment Tool-Modified (SPAT-M). Learner perceptions were explored through a post-clerkship survey and free-text reflections.

**Results:**

All 66 students completed the mandatory simulation curriculum; 46 (69.7%) completed the post-clerkship survey, narrowly below the predefined 70% threshold. Of 16 recordings screened, nine (56.3%) met technical criteria for SPAT-M analysis; seven (43.7%) were excluded for incomplete capture, audio failure, or poor image quality. Students reported high perceived NTS competence (median 4.0–4.5/5); faculty ratings of the nine usable recordings reflected competent-to-proficient performance (median 3.5–4.5). Situational awareness was the relatively lowest-rated domain and the most frequently reported area for development. Thematic analysis identified three themes: applying NTS in clinical practice, situational awareness as an area for development, and support for earlier longitudinal NTS integration.

**Conclusion:**

Integrating VAD into a mandatory emergency medicine clerkship was feasible, although survey completion narrowly missed the predefined threshold and technical limitations restricted structured assessment to 56.3% of recordings. Learners perceived VAD as supporting reflection on NTS performance. Further multi-institutional studies are needed to evaluate educational effectiveness and transfer to clinical practice.

## Introduction

Non-technical skills (NTS), including communication, teamwork, leadership, situational awareness, decision-making, and task management, are essential for safe and effective clinical practice.^1–6^ In high-acuity environments such as the emergency department, deficiencies in these skills may contribute to communication failures, impaired team performance, delayed escalation, and preventable adverse events.^2–4^,^6^ Despite increasing recognition of their importance, undergraduate medical education continues to emphasize biomedical knowledge and procedural competence, while NTS are often taught inconsistently or addressed implicitly.^7–10^ Structured behavioral marker systems for non-technical skills were first developed in anaesthesia,^11^ and have since been applied in intensive care^12^ and emergency medicine teams,^13^ as well as adapted for medical students.^7^,^8^ The relevance of NTS extends across acute-care specialties, including surgery^14^ and urology,^15^ underscoring their importance beyond emergency medicine alone.

Simulation-based medical education provides an established platform for experiential learning and deliberate practice of both technical and non-technical skills.^16–20^ By recreating realistic clinical scenarios in a safe educational environment, simulation enables learners to practice acute care decision-making, team communication, leadership behaviors, and task prioritization without risk to patients. Simulation also promotes reflection, feedback, and behavioral modification, which are central to professional development and experiential learning theory.^18^,^19^

Debriefing is a core component of simulation-based learning.^16^,^17^,^21^ It enables learners to examine clinical reasoning, decision-making, communication, team behaviors, and performance gaps in a structured manner. Video-assisted debriefing (VAD) allows learners to revisit complex team interactions after the immediate cognitive demands of the scenario have subsided. This retrospective review may reduce extraneous cognitive load, support more deliberate processing, and help learners connect observed behaviors with their consequences. VAD may therefore be particularly useful for NTS such as situational awareness, leadership, and communication, which can be difficult to appreciate fully in real time, consistent with complexity-based models of NTS learning.^22^

However, evidence regarding VAD for NTS development in undergraduate medical education remains limited. Undergraduate learners may find NTS difficult because they have less clinical experience managing multiple simultaneous demands, anticipating deterioration, and coordinating team activity under pressure. Previous VAD studies have often focused on short-term, elective, or research-specific interventions rather than routine clinical clerkships. The feasibility of embedding structured VAD within a mandatory undergraduate emergency medicine clerkship—including participation, technical reliability, and learner perceptions—remains insufficiently explored.

The primary aim was to evaluate the feasibility of integrating VAD into a mandatory emergency medicine clerkship. The secondary aims were to descriptively explore learner perceptions of VAD and to describe student-reported and faculty-observed NTS performance. Because the study had no baseline assessment, comparator group, or predefined performance thresholds, it was designed to generate preliminary, context-specific feasibility data rather than evidence of effectiveness.

## Materials and Methods

### Study Design and Setting

This convergent mixed-methods feasibility study^23^ was conducted within the mandatory emergency medicine clerkship for final-year medical students at Hamad General Hospital, Doha, Qatar. Simulation activities were delivered at the ITQAN Clinical Simulation and Innovation Center. The study combined descriptive quantitative assessment with qualitative analysis of learner reflections. Feasibility was defined a priori using three indicators: participation in the mandatory simulation curriculum ≥80%, post-clerkship survey completion ≥70%, and ≥50% of screened recordings meeting technical criteria for independent assessment. Because session attendance was compulsory, participation was treated as an implementation indicator rather than evidence of voluntary acceptability.

### Participants

All final-year medical students enrolled in the emergency medicine clerkship between September 2025 and April 2026 were eligible (N=66); therefore, no sampling or selective recruitment was undertaken. Participation in weekly simulation sessions was mandatory as part of the clerkship curriculum. Participation in the research survey and use of de-identified data for research were voluntary. Students with prior formal structured NTS training beyond the standard undergraduate curriculum were to be excluded; no students met this criterion.

### Educational Intervention

The 8-week emergency medicine clerkship included weekly 90-minute simulation sessions. Students participated in groups of 6–8 and managed 15-minute emergency scenarios, including ST-elevation myocardial infarction, anaphylaxis, asthma exacerbation, trauma resuscitation, and acute respiratory failure. Each scenario used predefined learning objectives and a standardized sequence of briefing, scenario delivery, and 30-minute faculty-led debriefing. Core clinical triggers and expected actions were specified in advance, while scenario progression was adjusted only in response to learner actions.

During debriefing, faculty addressed both clinical management and NTS, including communication, teamwork, leadership, situational awareness, decision-making, and task management. Approximately 5–8 minutes of selected playback was used within each 30-minute debriefing. Facilitators selected three predefined types of moments: the initial team response, a critical decision point, and a handover or task transition. Video was paused to invite learner reflection before faculty feedback, using an advocacy-inquiry approach.^24^ The six Simulation Performance Assessment Tool–Modified (SPAT-M) domains informed facilitator attention, although not every domain was necessarily discussed in equal depth during every session; emphasis depended on the behaviors observed.

This iterative cycle of simulation, video review, reflection, and feedback was repeated throughout the clerkship. The structured playback categories, facilitator prompts, and advocacy-inquiry approach were used to improve consistency across sessions.

### Video Recordings

Simulation scenarios were conducted during weekly Wednesday teaching sessions across three eight-week emergency medicine clerkship rotations, each comprising 22 final-year medical students. All scenarios were scripted and delivered using standardized learning objectives, assessment rubrics, and structured faculty feedback. Video-assisted debriefing followed each recorded scenario, and students provided written consent for recording through the simulation center.

During the study period, 16 simulation recordings were available: five from the first clerkship rotation, five from the second, and six from the third. All 16 recordings were screened for technical eligibility. Recordings were eligible for inclusion if they contained complete audio, clear visualization of all team members, and uninterrupted capture from the beginning to the end of the scenario. Seven recordings were excluded because they did not meet these criteria, including incomplete capture of team interactions (n = 4), audio failure (n = 2), and poor image quality (n = 1).

The remaining nine recordings met the eligibility criteria and were included in the SPAT-M analysis. These recordings were evenly distributed across the three clerkship rotations, with three recordings selected from each rotation to ensure balanced representation.

The students represented in the analyzed recordings belonged to the same cohort of 66 eligible students. However, because the post-clerkship questionnaire was completed anonymously, individual survey responses could not be linked to students appearing in the analyzed videos.

### Assessment Instruments

#### Faculty Assessment: Simulation Performance Assessment Tool–Modified (SPAT-M)

Simulation performance was assessed using the Simulation Performance Assessment Tool–Modified (SPAT-M) (Appendix A and Table A1), a behavioral assessment tool scored on a 5-point scale informed by Benner’s novice-to-expert framework, where 1 indicates novice performance and 5 indicates expert performance.^25^ The tool was adapted from established behavioral marker systems for non-technical skills assessment and contextualized for the local educational setting. The six assessed domains were situational awareness, decision-making, communication, teamwork and collaboration, leadership, and task management.

#### Student Self-Assessment Questionnaire

At the end of the clerkship, students completed a structured self-assessment questionnaire evaluating their perceived competence across the same six non-technical skills domains (Appendix B and Table B1). The questionnaire included 15 Likert-scale items rated on a 5-point scale, from 1 = novice to 5 = expert, and three open-ended questions exploring perceived strengths, areas for improvement, and reflections on the educational experience.

The questionnaire was developed by the study team based on established non-technical skills frameworks and reviewed by faculty with expertise in simulation-based education and medical education. Formal psychometric validation is ongoing; however, internal consistency for the 15 Likert-scale items in this sample was acceptable, with a Cronbach’s alpha of 0.87.

### Assessment Process and Acceptability Assessment

Two faculty assessors independently reviewed all included recordings several weeks after clerkship completion. Before formal rating, they reviewed the SPAT-M domains and scoring anchors and discussed sample behaviors to support a shared interpretation of the instrument. Assessors were blinded to student identity. Each recording was rated independently, and no consensus score replaced the original ratings.

Both assessors were board-certified emergency medicine consultants with 13 and 15 years of post-certification clinical experience, respectively, and at least 5 years of experience in simulation-based education. Both had prior experience teaching non-technical skills within the simulation curriculum; however, this was their first experience formally assessing recorded simulation performance using a structured instrument such as the SPAT-M.

Acceptability was explored indirectly through post-clerkship self-assessment responses and free-text reflections concerning the perceived educational value of simulation and VAD (Table 1). No separate validated acceptability instrument was used; therefore, these findings are reported as descriptive learner perceptions rather than a definitive measure of acceptability.

**Table 1:** Video-Assisted Debriefing Protocol

Component	Description
Timing	Video playback occurred immediately after the simulation scenario and before or during structured debriefing.
Duration	Approximately 5–8 minutes of selected playback per 30-minute debriefing.
Playback segments	Three key moments were selected: initial team response, critical decision point, and handover or task transition.
Facilitator prompt	Example: “Watch your team’s response in the first two minutes. What do you notice about how information was shared?”
Pause points	Video was paused at key moments to invite student reflection.
Debriefing approach	Faculty used an advocacy-inquiry approach, for example: “I noticed this behavior. What were you thinking at that moment?”
Student role	Students were encouraged to identify their own behaviors before receiving faculty feedback.

### Quantitative Data Analysis

Quantitative data were analyzed using IBM SPSS Statistics, version 29 (IBM Corp, Armonk, NY, USA). Likert-type data are summarized using medians and interquartile ranges. Two faculty assessors independently rated each included video across the six SPAT-M domains. Inter-rater reliability was calculated for the global SPAT-M score using a two-way mixed-effects Intraclass Correlation Coefficient (ICC) with an absolute-agreement model and was high in this sample (ICC=0.91; 95% CI, 0.85–0.95). The ICC was not calculated separately for individual domains and should therefore be interpreted as agreement on overall performance rather than domain-specific consistency.

Faculty ratings were derived from nine recordings, whereas student self-ratings came from 46 individual survey respondents. These datasets differ in both sample and unit of analysis; therefore, no direct statistical comparison was performed. Results are presented separately and descriptively.

### Qualitative Data Analysis

Free-text responses from 46 participants were analyzed using reflexive thematic analysis following Braun and Clarke’s six-phase framework.^26^ Coding and theme development were conducted independently by two researchers. Discrepancies were resolved through discussion and consensus with a third reviewer.

### Data Integration

Quantitative and qualitative findings were integrated during interpretation using a convergent approach. Integration focused on areas of convergence and divergence across student perceptions, faculty-observed performance, and qualitative themes, without treating the datasets as directly equivalent.

## Results

### Participant Flow and Feasibility Metrics

Among 66 eligible students, all 66 completed the mandatory simulation curriculum. Because attendance was compulsory, this finding reflects curricular reach rather than voluntary acceptability. Forty-six students completed the post-clerkship survey (69.7%), narrowly below the predefined feasibility threshold of ≥70%. Non-response reflected scheduling conflicts (n=12), declined participation (n=5), and incomplete responses (n=3).

For objective behavioral assessment, 16 simulation recordings were screened. Nine recordings (56.3%) met predefined technical inclusion criteria (complete audio, clear visualization of all team members, uninterrupted recording), exceeding the prespecified feasibility criterion of ≥50%. Seven recordings (43.7%) were excluded due to technical limitations: incomplete capture of team interactions (n=4), audio failure (n=2), and poor image quality (n=1). Each included recording was independently assessed by two faculty raters, generating 18 ratings.

**Figure 1:**
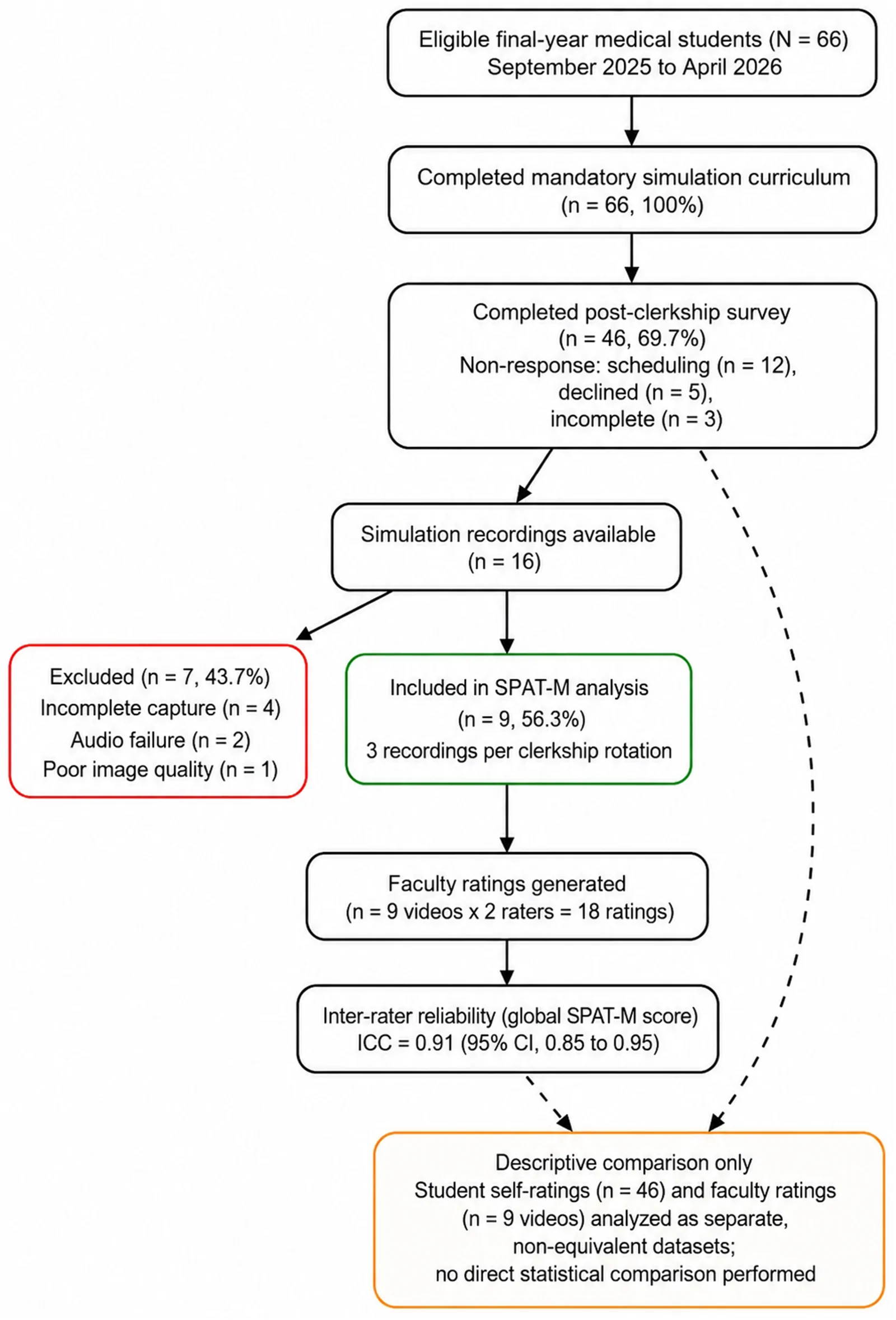
Participant and Data Flow. This conceptual illustration was generated with assistance from a large language model (ChatGPT, GPT-4o) and subsequently reviewed, edited, and scientifically validated by the authors, who assume full responsibility for its content and accuracy.

**Abbreviations**: SPAT-M, Simulation Performance Assessment Tool–Modified; ICC, intraclass correlation coefficient; CI, confidence interval; N, total eligible participants; n, number of participants or recordings within each analysis stage.

Inter-rater reliability for the global SPAT-M score between the two faculty assessors was high (ICC=0.91; 95% CI, 0.85–0.95), though this should be interpreted alongside the small number of included recordings (n=9) and does not reflect domain-specific agreement (Figure 1 and Table 2).

**Table 2:** Participant Flow and Inclusion

Stage	n	%	Notes
Eligible students	66	100	Final-year medical students
Completed simulation curriculum	66	100	Mandatory clerkship sessions
Completed post-clerkship survey	46	69.7	Self-assessment respondents
Videos screened	16	–	Available recordings
Videos included in SPAT-M analysis	9	56.3	7 recordings excluded due to technical limitations
Faculty raters	2	–	ICC = 0.91

**Note**: Flow of participants and video recordings through the study.

**Abbreviations**: ICC, intraclass correlation coefficient; SPAT-M, Simulation Performance Assessment Tool-Modified.

### Self-Reported Non-Technical Skills (Student Survey, N=46)

Students reported high perceived competence across the six NTS domains. However, self-assessment of behavioral competence may not closely correspond to observed performance, and the faculty dataset represented only nine team videos rather than the 46 individual survey respondents. The two datasets therefore provide complementary but non-equivalent perspectives. VAD may support metacognitive reflection by allowing learners to review complex interactions after the event, but this study did not test whether such reflection translated into measurable improvement (Table 3).

**Table 3:** Self-Assessed Non-Technical Skills Scores (N=46)

Domain	Median	IQR	Benner Level
Situational awareness	4.0	4.0–5.0	Proficient
Decision-making	4.0	3.5–4.0	Proficient
Communication	4.5	4.0–5.0	Proficient–Expert
Teamwork and collaboration	4.5	4.0–5.0	Proficient–Expert
Leadership	4.0	4.0–5.0	Proficient
Task management	4.5	4.0–5.0	Proficient–Expert

**Notes**: Student self-ratings of perceived non-technical skills competence at the end of the 8-week clerkship. Scores are based on a 5-point scale derived from Benner’s novice-to-expert framework (1=novice, 2=advanced beginner, 3=competent, 4=proficient, 5=expert).

**Abbreviation**: IQR, interquartile range.

### Faculty-Rated Performance (SPAT-M, n=9 Videos, 18 Ratings)

Faculty assessment demonstrated competent-to-proficient performance across all NTS domains (median scores 3.5–4.5). Task management received the highest median faculty rating (4.5, IQR 4.0–5.0), while situational awareness received the lowest median score (3.5, IQR 3.0–4.0). Decision-making, communication, teamwork and collaboration, and leadership each had median scores of 4.0, though teamwork showed wider variability (IQR 3.0–5.0) (Table 4).

**Table 4:** Faculty-Rated SPAT-M Scores (n=9 Videos; 18 Ratings)

Domain	Median	IQR	Benner Level
Situational awareness	3.5	3.0–4.0	Competent–Proficient
Decision-making	4.0	3.0–4.0	Proficient
Communication	4.0	3.0–4.0	Proficient
Teamwork and collaboration	4.0	3.0–5.0	Proficient–Expert
Leadership	4.0	3.0–5.0	Proficient
Task management	4.5	4.0–5.0	Proficient–Expert

**Notes**: Faculty ratings of student non-technical skills performance based on independent review of video-recorded simulation sessions. Scores are based on a 5-point scale derived from Benner’s novice-to-expert framework (1=novice, 2=advanced beginner, 3=competent, 4=proficient, 5=expert). Each of the nine videos was rated by two independent faculty assessors, for a total of 18 ratings.

**Abbreviations**: IQR, interquartile range; SPAT-M, Simulation Performance Assessment Tool-Modified.

### Descriptive Contextualization of Student and Faculty Ratings

Student self-ratings and faculty ratings are presented as separate descriptive datasets because they were derived from different samples and units of analysis. Student self-ratings came from 46 respondents, whereas faculty ratings represented nine recorded team performances. No direct statistical comparison was undertaken, and apparent differences should not be interpreted as agreement or disagreement between the same learners and assessors.

### Qualitative Findings

Thematic analysis of free-text responses from 46 students identified three major themes and two cross-cutting subthemes. Students described simulation as helping them apply NTS in clinical practice, identified situational awareness as a relative area for further development, and supported earlier longitudinal integration of NTS training (Table 5).

**Table 5:** Representative Student Free-Text Responses Mapped to Themes

Theme/Subtheme	Representative Quote	Interpretation
Transition from theory to practice	“Communication: I developed this skill during my emergency clerkship”.	Simulation and clerkship exposure helped students recognize communication as a practical clinical skill.
Situational awareness gap	“I need to improve awareness of the clinical environment”.	Students recognized the need to move from reactive task completion toward proactive anticipation.
Support for earlier integration	“Decision making. I think this would improve with time, experience, and knowledge”.	Students viewed NTS as developmental competencies requiring repeated practice.
Psychological safety	Students openly identified areas for improvement.	Learners appeared willing to acknowledge performance gaps.
Video-assisted reflection	Students identified specific behaviors for improvement (eg, anticipating deterioration, preparing earlier).	VAD may support concrete, behavior-focused reflection.

**Notes**: Representative free-text responses from 46 students, mapped to the three main themes and two cross-cutting subthemes identified through reflexive thematic analysis. Each quote is accompanied by an interpretive summary.

### Theme 1: Transition from Theoretical Knowledge to Applied Clinical Practice

Students reported that NTS became more meaningful when applied during simulation and clinical encounters. Communication, teamwork, and interprofessional interaction were commonly described as skills developed through direct participation in the emergency medicine clerkship.

Communication: I developed this skill during my emergency clerkship by communicating with other specialties like surgery and other health members including nurses, residents and consultants, and gained knowledge and enhanced my clinical skills.

This theme suggests that simulation and workplace-based exposure helped students recognize NTS as practical behaviors required for safe emergency care.

### Theme 2: Situational Awareness as a Relative Area for Further Development

Situational awareness was the most frequently identified area for improvement, reported by 20 of 46 respondents (43.5%). Students described the need to monitor the clinical environment more proactively, anticipate deterioration, and prepare interventions in advance.

I need to improve awareness of the clinical environment, anticipating potential deterioration and preparing necessary interventions in advance.

This qualitative theme converged with the faculty dataset, in which situational awareness had the relatively lowest median score (3.5), while still reflecting competent-to-proficient performance.

### Theme 3: Support for Earlier Curriculum Integration

Most respondents supported earlier integration of NTS training into the medical curriculum. Students appeared to view these skills as developmental competencies requiring repeated exposure, practice, and feedback.

Decision making. I think this would improve with time, experience, and knowledge.

This suggests that learners perceived final-year clerkships as consolidation phases rather than the ideal point for first exposure to NTS training.

### Cross-Cutting Subthemes

Two cross-cutting subthemes were identified. First, responses suggested psychological safety^27^ because learners openly acknowledged areas for improvement. Second, VAD appeared to support specific, behavior-focused reflection, including anticipating deterioration, preparing earlier, and improving environmental awareness. These interpretations are descriptive and should not be taken as evidence of causal educational effect.

## Discussion

This mixed-methods feasibility study generated preliminary, context-specific data on embedding structured NTS teaching and VAD within a mandatory final-year emergency medicine clerkship. All students completed the compulsory simulation curriculum, 69.7% completed the post-clerkship survey, and 56.3% of screened recordings were technically usable for structured faculty assessment. The survey response rate narrowly missed the predefined threshold, and almost half of the recordings could not be assessed because of technical limitations. These findings indicate that curricular delivery was achievable in this setting while also identifying important implementation constraints.

Student-reported and faculty-observed NTS results were descriptive and came from different datasets. They should not be interpreted as evidence that VAD improved performance or as direct comparisons between the same individuals. The study’s principal contribution is implementation-focused: it describes how VAD was incorporated into routine clerkship teaching and highlights technical, measurement, and data-flow issues that future studies should address.

### Feasibility and Acceptability

The 100% participation rate primarily reflects the mandatory clerkship structure and should not be interpreted as voluntary acceptability. The 69.7% survey completion rate narrowly missed the 70% a priori threshold. In addition, only 56.3% of screened recordings met technical criteria for structured assessment. Together, these results suggest partial feasibility rather than unequivocally successful implementation. Programs considering VAD should plan for standardized camera placement, pre-session audiovisual checks, backup audio capture, and clear procedures for identifying technically adequate recordings. The 43.7% technical failure rate observed in this study suggests that VAD cannot be reliably implemented without dedicated audiovisual support, standardized camera placement protocols, and mandatory pre-session equipment checks. Institutions with limited simulation resources or technical staff should carefully weigh these infrastructure requirements against the anticipated educational benefits before committing to VAD integration.

Students reported high perceived competence across all NTS domains (median self-ratings 4.0–4.5, proficient to proficient-expert). However, self-assessments of behavioral competencies correlate only modestly with observed performance,^28^ and student ratings were generally higher than faculty ratings. This discrepancy may reflect VAD’s developmental role in promoting metacognitive insight, aligning with experiential learning theory where reflection on concrete experience drives behavioral change.^18^,^24^

### Situational Awareness as a Relative Area for Further Development

Situational awareness was the relatively lowest-rated faculty domain, with a median of 3.5, while still corresponding to competent-to-proficient performance. It was also the most frequently reported area for improvement in student reflections. Without baseline data, a comparator group, predefined thresholds, or an external benchmark, it should not be described as an objective deficit or the “most difficult” domain. Rather, the convergence of descriptive findings identifies it as a potential priority for further curriculum development.

Situational awareness requires learners to perceive relevant information, interpret its meaning, and anticipate likely clinical developments. Undergraduate learners may find this particularly demanding because they have limited experience coordinating multiple tasks and recognizing evolving patterns in high-acuity settings. Repeated exposure across progressively complex scenarios may therefore be more appropriate than concentrating this training solely in the final year.

### Support for Earlier Curriculum Integration

A strong qualitative theme was student support for earlier NTS integration into the medical curriculum. Students viewed final-year clerkships as consolidation phases rather than the ideal point for first exposure. One student commented: “Decision making…would improve with time, experience, and knowledge”. This aligns with NTS as developmental competencies requiring repeated exposure across training years.^2^,^6^,^9^,^10^

From a curriculum design perspective, this supports a longitudinal or spiral model, where NTS concepts are introduced early (eg, pre-clinical simulation) and revisited with increasing complexity. The final-year clerkship would then serve as consolidation rather than first exposure.

### Integration with Existing Literature

Embedding VAD within a compulsory, high-volume clerkship extends prior work conducted mainly in short-term, elective, or research-specific settings. The behavior-focused reflections described by learners are consistent with literature suggesting that video review can support self-observation and actionable feedback. However, because this study did not compare VAD with verbal debriefing alone,^29^ its specific added educational value remains uncertain. The findings are best viewed as implementation data that can inform the design of controlled undergraduate studies.

### Limitations and Future Research Directions

This study has several limitations. Its single-group design, absence of baseline assessment, lack of a comparator, and no long-term follow-up prevent conclusions about educational effectiveness, developmental change, or transfer to clinical practice.

The objective assessment sample was small: only nine of 16 screened recordings met technical criteria. This may have introduced selection bias and limits the precision and generalizability of faculty ratings. Future studies should prospectively document the full recording pathway and use standardized audiovisual procedures.

The student questionnaire was developed by the study team and has not undergone full psychometric validation. Acceptability was assessed indirectly through survey responses and reflections rather than through a dedicated validated measure. Participant characteristics such as prior simulation exposure, previous emergency medicine experience, and earlier informal NTS teaching were not systematically collected and may have influenced the high ratings.

Finally, this was a single-institution study conducted in one emergency medicine clerkship. Findings may not generalize to institutions with different curricula, simulation resources, faculty expertise, or learner populations. Multi-institutional studies are needed to evaluate feasibility, effectiveness, and implementation requirements across different educational settings.

## Conclusion

This study provides preliminary, context-specific feasibility data on integrating VAD into a mandatory emergency medicine clerkship. Curricular delivery was achievable, but the survey response rate narrowly missed the predefined threshold and 43.7% of screened recordings were technically unusable. Learners described VAD as supporting reflection, and situational awareness emerged as a relative area for further development rather than an objective performance deficit. Controlled, multi-institutional studies with standardized recording procedures and validated outcome measures are needed to evaluate effectiveness and transfer to clinical practice. Pending such studies, institutions implementing VAD should prioritize standardized audiovisual protocols and consider introducing NTS training earlier in the undergraduate curriculum, with final-year clerkships serving as consolidation rather than first exposure.

## Data Availability

De-identified data are available from the corresponding author upon reasonable request, subject to institutional approvals.

## Artificial Intelligence (AI) Declaration for Figure Preparation

Figure 1 was created with assistance from a large language model (ChatGPT, GPT-4o). The resulting image was subsequently reviewed, revised, and verified by the authors. The authors confirm that the originality and scientific accuracy of the figure content have been independently assessed and validated. The authors have reviewed the terms of use of the AI tool and confirm that the generated image is suitable for academic publication and that they have the right to publish the image. The authors take full responsibility for the integrity, accuracy, and interpretation of all content presented in the figure.

## References

[1] Flin R, O’Connor P, Crichton M. *Safety at the Sharp End: A Guide to Non-Technical Skills*. London: CRC Press; 2008. doi:10.1201/9781315607467

[2] Gordon M, Darbyshire D, Baker P. Non-technical skills training to enhance patient safety: a systematic review. *Med Educ*. 2012;46(11):1042–11. doi:10.1111/j.1365-2923.2012.04343.x 23078681

[3] Leonard M, Graham S, Bonacum D. The human factor: the critical importance of effective teamwork and communication in providing safe care. *Qual Saf Health Care*. 2004;13 Suppl 1:i85–i90. doi:10.1136/qshc.2004.010033 15465961 PMC1765783

[4] Hull L, Arora S, Aggarwal R, Darzi A, Vincent C, Sevdalis N. The impact of nontechnical skills on technical performance in surgery: a systematic review. *J Am Coll Surg*. 2012;214(2):214–230. doi:10.1016/j.jamcollsurg.2011.10.016 22200377

[5] Riaz S, Naemi R. How the training level has influenced clinical year medical students’ approach towards learning and practising non-technical skills. *J Pract Teach Learn*. 2024;21(3):6–26. doi:10.1921/jpts.v20i3.2035

[6] Prydz K, Dieckmann P, Musson D, Wisborg T. The development of a tool to assess medical students’ non-technical skills - The Norwegian medical students’ non-technical skills (NorMS-NTS). *Med Teach*. 2023;45(5):516–523. doi:10.1080/0142159X.2022.2140034 36345232

[7] Hamilton AL, Kerins J, MacCrossan MA, Tallentire VR. Medical Students’ Non-Technical Skills (Medi-StuNTS): preliminary work developing a behavioural marker system for the non-technical skills of medical students in acute care. *BMJ Simul Technol Enhanc Learn*. 2018;5(3):130–139. doi:10.1136/bmjstel-2018-000310 PMC8936547 35514943

[8] Phillips EC, Smith SE, Clarke B, et al. Validity of the Medi-StuNTS behavioural marker system: assessing the non-technical skills of medical students during immersive simulation. *BMJ Simul Technol Enhanc Learn*. 2020;7(1):3–10. doi:10.1136/bmjstel-2019-000506 PMC8936660 35521075

[9] Khaw SC, Chung L, Fancourt N. Integrating non-technical skills into undergraduate medical simulation: a scoping review and thematic analysis of current practices. *Adv Simul*. 2025;10(1):49. doi:10.1186/s41077-025-00377-9 PMC12542256 41121410

[10] Riaz S, Naemi R. An investigation into when and how to train medical students for the most effective learning of non-technical skills: a qualitative study. *J Pract Teach Learn*. 2024;22(1–2):154–177. doi:10.1921/jpts20242374

[11] Fletcher G, Flin R, McGeorge P, Glavin R, Maran N, Patey R. Rating non-technical skills: developing a behavioural marker system for use in anaesthesia. *Cogn Technol Work*. 2004;6(3):165–171. doi:10.1007/s10111-004-0158-y

[12] Reader T, Flin R, Lauche K, Cuthbertson BH. Non-technical skills in the intensive care unit. *Br J Anaesth*. 2006;96(5):551–559. doi:10.1093/bja/ael067 16567346

[13] Cant RP, Porter JE, Cooper SJ, Roberts K, Wilson I, Gartside C. Improving the non-technical skills of hospital medical emergency teams: the Team Emergency Assessment Measure (TEAM™). *Emerg Med Australas*. 2016;28(6):641–646. doi:10.1111/1742-6723.12643 27474369

[14] Yule S, Flin R, Paterson-Brown S, Maran N. Non-technical skills for surgeons in the operating room: a review of the literature. *Surgery*. 2006;139(2):140–149. doi:10.1016/j.surg.2005.06.017 16455321

[15] Griffin C, Aydın A, Brunckhorst O, et al. Non-technical skills: a review of training and evaluation in urology. *World J Urol*. 2020;38(7):1653–1661. doi:10.1007/s00345-019-02920-6 31529246 PMC7303051

[16] McGaghie WC, Issenberg SB, Petrusa ER, Scalese RJ. A critical review of simulation-based medical education research: 2003-2009. *Med Educ*. 2010;44(1):50–63. doi:10.1111/j.1365-2923.2009.03547.x 20078756

[17] Issenberg SB, McGaghie WC, Petrusa ER, Lee Gordon D, Scalese RJ. Features and uses of high-fidelity medical simulations that lead to effective learning: a BEME systematic review. *Med Teach*. 2005;27(1):10–28. doi:10.1080/01421590500046924 16147767

[18] Kolb DA. *Experiential Learning: Experience as the Source of Learning and Development*. Englewood Cliffs, NJ: Prentice-Hall; 1984. ISBN: 978-0132952613.

[19] Knowles MS. *The Adult Learner: A Neglected Species*. 4th ed. Houston: Gulf Publishing; 1990.

[20] Lavoie P, Michaud C, Bélisle M, et al. Learning theories and tools for the assessment of core nursing competencies in simulation: a theoretical review. *J Adv Nurs*. 2018;74(2):239–250. doi:10.1111/jan.13416 28815750

[21] Decker S, Sapp A, Bibin L, et al. The impact of simulation debriefing process on learning outcomes: an umbrella review. *Clin Simul Nurs*. 2025;101:101715. doi:10.1016/j.ecns.2025.101715

[22] Gordon M, Box H, Farrell M, Stewart A. Non-technical skills learning in healthcare through simulation education: integrating the SECTORS learning model and complexity theory. *BMJ Simul Technol Enhanc Learn*. 2015;1(2):67–70. doi:10.1136/bmjstel-2015-000047 PMC8936904 35520017

[23] Katz-Buonincontro J. Convergent mixed methods designs. In: *How to Mix Methods: A Guide to Sequential, Convergent, and Experimental Research Designs*. Washington, DC: American Psychological Association;2024:73–82. doi:10.1037/0000404-005

[24] Gasteratos K, Daniels B, Gebhart SJ, et al. Three-phase video-assisted multidisciplinary team debriefing in high-fidelity blast simulation through the advocacy and inquiry method. *Plast Reconstr Surg*. 2024;154(2):453–463. doi:10.1097/PRS.0000000000011070 37734003

[25] Benner P. *From Novice to Expert: Excellence and Power in Clinical Nursing Practice*. Commemorative ed. Upper Saddle River, NJ: Prentice Hall; 2000. ISBN: 978-0130325228.

[26] Braun V, Clarke V. Using thematic analysis in psychology. *Qual Res Psychol*. 2006;3(2):77–101. doi:10.1191/1478088706qp063oa

[27] Rudolph JW, Raemer DB, Simon R. Establishing a safe container for learning in simulation: the role of the presimulation briefing. *Simul Healthc*. 2014;9(6):339–349. doi:10.1097/SIH.0000000000000047 25188485

[28] Kruger J, Dunning D. Unskilled and unaware of it: how difficulties in recognizing one’s own incompetence lead to inflated self-assessments. *J Pers Soc Psychol*. 1999;77(6):1121–1134. doi:10.1037/0022-3514.77.6.1121 10626367

[29] Rueda-Medina B, Reina-Cabello JC, Buendía-Castro M, et al. Effectiveness of video-assisted debriefing versus oral debriefing in simulation-based interdisciplinary health professions education: a randomized trial. *Nurse Educ Pract*. 2024;75:103901. doi:10.1016/j.nepr.2024.103901 38277804

